# A SARS Method for Reliable Spectrum Sensing in Multiband Communication Systems

**DOI:** 10.1109/TSP.2011.2165060

**Published:** 2011-08-15

**Authors:** Bashar I. Ahmad, Andrzej Tarczynski

**Affiliations:** University of Westminster 115 New Cavendish StreetLondon U.K. W1W 6UW

**Keywords:** Fourier transform, hypothesis testing, nonuniform sampling, spectral analysis, spectrum sensing

## Abstract

This paper introduces a novel method of spectrum sensing in communication systems that utilizes nonuniform sampling in conjunction with a suitable spectral analysis tool. It is referred to here as spectral analysis for randomized sampling (SARS). Owing to the deployment of nonuniform sampling, the proposed technique can accomplish the sensing task by using sampling rates well below the ones demanded by uniform-sampling-based digital signal processing (DSP). The effect of the cyclostationary nature of the incoming digital communication signal on the adequacy of the adopted periodogram-type estimator for the spectrum sensing operation is addressed. The statistical characteristics of the estimator are presented. General reliability conditions on the length of the required signal observation window, i.e., sensing time, for a chosen sampling rate or vice versa are provided amid a sought system performance. The impact of the presence of noise and processing transmissions with various power levels on the derived dependability recommendations is given. The analytical results are illustrated by numerical examples. This paper establishes a new framework for efficient spectrum sensing where considerable savings on the sampling rate and number of processed samples can be attained.

## I. Introduction

Spectrum sensing entails scanning parts of the radio spectrum in search for a meaningful activity, such as a transmission or the occurrence of an event. Its techniques have lately received notable attention due to their crucial role in the emerging cognitive radio (CR) technology, i.e., by unveiling spectral holes for opportunistic spectrum access. Several reviews on the topic exist, e.g., [1]–[4]. This adds to the plethora of spectrum sensing application areas such as surveillance/interception [5], astronomy [6] and [7] as well as seismology [8]. Sensing methods that rely on nonparametric spectral analysis/estimation are regarded as efficient and adequate candidates for monitoring a wide frequency range consisting of a number of predefined nonoverlapping spectral subbands, without a priori knowledge of the signal's characteristics [1]–[3]. Such methods have clear advantages over single-band oriented ones, for instance those based on matched filtering or feature detecting that require the separation of the individual transmissions typically by tunable bandpass filtering [3]. In this paper, a multiband spectrum sensing technique that uses a periodogram-type spectral analysis tool to estimate the spectrum of the incoming signal from a finite set of its samples is adopted. This means has retained its popularity in several spectrum sensing studies, e.g., [9]–[12].

When uniform-sampling-based DSP is deployed, the sampling rate of the sensing device should exceed at least twice the total bandwidth of the monitored frequency range regardless of the spectrum occupancy [13]. Failing to do so results in spectrum aliasing and irresolvable detection problems. In the event of examining wide bandwidths such a constraint can pose a challenge to the system designer where a high sampling rate, high speed signal processing and treating large quantities of data are required [2], [3]. Here, we demonstrate that we can detect the active spectral subbands by the suitable use of arbitrarily low rate nonuniform sampling and appropriate processing of the signal—a methodology referred to as digital alias-free signal processing (DASP). A few monographs, e.g., [14] and [15], give an overview on the topic. Operating at low sampling rates can exploit the sensing device resources (such as power) more efficiently and/or avoid the possible need for a high-cost fast hardware. The main focus of this paper is to explore the possibility and benefits of employing the DASP methodology to conduct reliable detection in wideband communication systems.

### A. Related Work

Lomb-Scargle periodogram in [6] and [7] is one of the oldest and most popular tools for spectral analysis of nonuniformly sampled data. Nevertheless, new techniques keep unfolding such as [16] and a review is given in [17]. While the aforementioned methods deal with arbitrary nonuniform sampling, the approach proposed here relies on the ability of the user to prescribe the positions of the sampling instants, i.e., randomized sampling and hence SARS. Spectral analysis of randomized sampling schemes for deterministic signals was studied in [18]–[20]. In this paper, the processed signal is assumed to be a random cyclostationary/nonstationary process. The earliest papers on DASP, e.g., [21] and [22], tackled SARS with the aim of estimating the signal's power spectral density (PSD) of wide sense stationary (WSS) signals; however the predicament of the estimators' accuracies for a finite number of samples was not resolved. This issue was partially addressed by Masry in [23] where asymptotic accuracy measures were given, i.e., when the number of samples and signal observation window tend to infinity. In this study, the characteristics and spectrum sensing capabilities of a spectral analysis method that uses a finite set of samples captured at low rate are investigated. The emphasis of the paper is therefore on reliable spectrum sensing rather than on estimating the exact PSD of the processed signal.

In [24], we investigated spectrum sensing of WSS signals using SARS where transmissions over the system subbands are presumed to be of equal power levels. Here, digital communication signals, which are modeled as cyclostationary processes, are analyzed and the scenario where the conveyed transmissions are of different power levels, e.g., due to the propagation channel gain, is considered. The reliability of detection is expressed in terms of the widely embraced receiver operating characteristics (ROC) in lieu of a general metric, i.e., Chebychev's inequality parameter as in [24]. Circumventing the nonstationary nature of the communication signals either via phase randomization, e.g., [25] and [26], or assuming pseudostationarity within a short signal time window, as in [4] and [12], is the common approach adopted in the literature. In [27], Gardner exposed the defects of such practices in an attempt to correct any errors incurred. In this paper, the effect of the signal's nonstationarity is acknowledged and its repercussions on the conducted spectral analysis are evaluated. Necessary steps are taken to appropriate the employed spectrum estimator to the studied problem, i.e., spectrum sensing and not PSD estimation. This includes alias-free sampling, windowing and estimate averaging.

### B. Contribution

We introduce a noncooperative multiband spectrum sensing method based on the spectral analysis of the incoming signal from a finite set of its nonuniformly distributed noisy samples. Compared to the classical uniform-sampling-based DSP, the proposed approach can offer substantial savings on the sampling rates and number of processed samples. Within this framework, we present the statistical characteristics of a periodogram-type estimator that uses the total random sampling (TRS) scheme. The impact of the cyclostationary/nonstationary nature of the processed signal on SARS is studied. A spectral fragment within each transmission band, referred to as the “guarded region” where the estimator serves as a suitable sensing tool independent of the position of the time analysis window, is identified. Additionally, a phenomenon exhibited by abrupt increases in the estimator's standard deviation at certain frequency points for some modulation schemes is unveiled.

The sensing reliability is defined in terms of the probability of detection and false alarm for each of the monitored subbands. It is demonstrated that these probabilities are closely related to the average sampling rate, sensing time, spectrum utilization, relative powers of active subbands and signal to noise ratio. We provide a lower limit on the first two parameters from the latter list such that the reliability of spectrum sensing is guaranteed; it represents a means to assess the trade-off between the required sampling rate and the sensing time. The advantages of the introduced technique over the conventional uniform sampling ones are discussed.

The paper is organized as follows. In Section II, the detection problem is formulated, the undertaken approach is detailed and the handled class of communication signals is outlined. The statistical characteristics of the deployed estimator are examined and ways to restrain its possible inaccuracies are highlighted in Section III. In Section IV, reliability recommendations are developed and the benefits of the employed detector over uniform-sampling-based ones are exposed. Numerical examples are shown in Section V to illustrate the proposed method. Finally, conclusions are drawn in Section VI.

## II. Wideband Spectrum Sensing

### A. Problem Formulation and Sensing Technique

Consider a communication system operating over $L$ narrow nonoverlapping spectral subbands, each of them is of width $B_{C}$. The total single-sided bandwidth that needs to be monitored by the system is $B=LB_{C}$. The positions of all the subbands are known. The maximum number of simultaneously active subbands at any particular point in time is $L_{A} \le L$, i.e., the joint bandwidth of the active subbands never exceeds $B_{A}=L_{A}B_{C}$. The incoming multiband signal consists of an unknown number of concurrently active subbands denoted by $M$ where $M \le L_{A}$ and is given by

$$x(t) = \sum\limits_{m = 1}^{M} {{x_{m}}(t)} = \sum\limits_{m = 1}^{M} {{x_{T,m}}(t) \ast {h_{m}}(t)} .\eqno{\hbox{(1)}}$$

The symbol “∗” represents the convolution operation whereas $x_{T,m}(t)$ and $h_{m}(t)$ are the transmitted signal over the $m$th active subband and the impulse response of its propagation channel, respectively. The captured samples of $x(t)$ are contaminated with additive white Gaussian noise (AWGN) with variance $\sigma_{n}^{2}$ and are defined by $y(t_{n})=x(t_{n})+n(t_{n})$. Our objective is to devise a method that is capable of scanning the overseen bandwidth $B$ and identifying the active subbands. The algorithm should operate at sampling rates significantly lower than $2B$ which is the minimum rate (not always achievable) that could be used when uniform sampling is deployed [13].

Unlike methods that employ spectral analysis to estimate the subbands energy/power, e.g., classical energy detectors [9]–[11], the sensing procedure for each spectral subband comprises two steps: 1) estimating the magnitude of the signal spectrum at selected frequency point(s); and 2) comparing the magnitude(s) with precalculated threshold(s). Having a spectrograph that is relatively smooth would permit assessing fewer frequency points per system subband to determine its status. Here, we seek to inspect one frequency point per subband, i.e., $L$ spectral points are calculated. The tackled sensing problem can be formulated as a conventional detection problem described by the following binary hypothesis testing

$$\eqalignno{{H_{0,k}}:& \quad {{{\mathhat X}_{e}}({f_{k}}) < {\gamma _{k}}} \cr {H_{1,k}}:& \quad {{\mathhat X}_{e}}({f_{k}}) \ge {\gamma _{k}} \qquad {k = 1,2, \ldots L} &{\hbox{(2)}}}$$

where ${\mathhat X}_{e}(f)$ is the estimated magnitude spectrum and $\gamma_{k}$ is the threshold. Whereas, $H_{0,k}$ hypothesis signifies the absence of an activity in $k$th subband and $H_{1,k}$ depicts the presence of an activity. The frequency points $\{f_{k}\}_{k=1}^{L}$ are placed at the center of the subbands as shown in Section III. We emphasize that SARS aims at estimating a detectable frequency representation of the received signal and not its PSD. The latter is defined as the Fourier transform (FT) of the signal's autocorrelation function [25].

### B. Signal Model

Let $x_{T,k}(t)$ be the continuous-time signal transmitted over one of the system active subbands by a communication source that deploys a linear digital modulation scheme. It can be expressed by: $x_{T,k}(t)=x_{i,k}(t)+x_{q,k}(t)$ where $x_{i,k}(t)=i_{k}(t) \cos (2 \pi f_{C,k}t)$ and $x_{q,k}(t)=q_{k}(t) \cos (2 \pi f_{C,k}t + 0.5 \pi)$. The in-phase $i_{k}(t)$ and quadrature $q_{k}(t)$ components are baseband signals given by: $i_{k}(t)= {\sum_{n=-\infty}^{+ \infty}} a_{n,k}p_{i,k}(t+nT_{S,k})$ and ${q_k}(t) = \sum_{n = - \infty }^{+ \infty } {{b_{n,k}}{p_{q,k}}(t + n{T_{S,k}})}$, respectively, where ${f_{C,k}}$ is the carrier frequency of the $k$th active subband and ${f_{S,k}} = {1/T_{S,k}}$ is its baud rate. The coefficients ${\left\{{{a_{n,k}}} \right\}_{n \in \BBZ}}$ and ${\left\{{{b_{n,k}}} \right\}_{n \in \BBZ}}$ are the transmitted symbols; they are zero mean independent and identically distributed (i.i.d.) random variables with variances of ${\sigma _{a,k}^2}$ and ${\sigma _{b,k}^2}$. The impulse response of the baseband shaping filters in the in-phase and quadrature branches are ${p_{i,k}}(t)$ and ${p_{q,k}}(t)$, respectively. Hence the incoming signal can be modeled as

$${x_{k}}(t) = \sum\limits_{n = - \infty }^{+ \infty } {{a_{n,k}}{s_{i,k}}(t,n)} + \sum\limits_{n = - \infty }^{+ \infty } {{b_{n,k}}{s_{q,k}}(t,n)} \eqno{\hbox{(3)}}$$

where ${s_{i,k}}(t,n) = \left[{{p_{i,k}}(t + n{T_{S,k}})\cos (2\pi {f_{C,k}}t)} \right] \ast {h_k}(t)$ and ${s_{q,k}}(t,n) = \left[{{p_{q,k}}(t + n{T_{S,k}})\cos (2\pi {f_{C,k}}t + 0.5\pi)} \right] \ast {h_k}(t)$.

It is in the interest of the forthcoming analysis to find certain first and second order moments of the processed signal. It can be easily checked that $E\left[{{x_k}(t)} \right] = 0$. Since ${a_{n,k}}$ and ${b_{n,k}}$ are independent, it is clear that $E\left[{{x_{i,k}}({t_1}){x_{q,k}}({t_2})} \right] = 0$. As a result, the autocorrelation function of (3) is

$$\displaylines{{R_{X,k}}(t,t + \tau) = {\sigma _{a,k}^{2}}\sum\limits_{n = - \infty }^{+ \infty } {{s_{i,k}}(t,n){s_{i,k}}(t + \tau ,n)} \hfill\cr\hfill + {\sigma _{b,k}^{2}} \sum\limits_{n = - \infty }^{+ \infty } {{s_{q,k}}(t,n){s_{q,k}}(t + \tau ,n)} \quad{\hbox{(4)}}}$$

whereas ${R_X}(t,t + \tau) = \sum_{m = 1}^{M }{{R_{X,m}}(t,t + \tau)}$ is for the $M$ independent simultaneously active transmissions. Noting that (4) is time-varying, such processes are commonly regarded as wide sense cyclostationary including the cases when the symbol period is not an integer multiple of the carrier period [27].

## III. Statistical Characteristics of SARS

The adopted total random sampling is an alias-free sampling scheme whose behavior was investigated in [18] and [19]. Its sampling instants $\left\{{t_n} \right\}$ are i.i.d. random variables with a probability distribution function (pdf) $p(t)$. Here, we consider $p(t) = {1 / {T_0}}$ for $t \in {{\cal T}_r}$ and zero elsewhere such that ${{\cal T}_r} = \left[{{{\zeta}_r},{{\zeta}_r} + {T_0}} \right]$ is the time analysis window. The deployed estimator of a detectable frequency representation of the incoming signal is given by

$${X_{e}}({{\zeta}_{r}},f) = {N \over {(N - 1)\mu}}{\left\vert {{{{T_{0}}} \over N}\sum\limits_{n = 1}^{N} {y({t_{n}})w({t_{n}}){e^{- j2\pi f{t_{n}}}}} } \right\vert^{2}} \eqno{\hbox{(5)}}$$

where ${\left\{{t_n} \right\}_{n = 1}^{N}}$ are the TRS sampling instants chosen inside ${\cal {T}}_{r}$, $w(t)$ is the windowing function, $N$ is the number of the collected noisy signal samples ${\left\{{y({t_n})} \right\}_{n = 1}^{N}}$ and $\mu = \int_{{{\zeta}_r}}^{{{\zeta}_r} + {T_0}} {{w^2}(t)dt}$. Typically, a $K$ number of ${X_e}({{\zeta}_r},f)$ estimates are averaged to improve its performance, i.e., ${\sum_{r = 0}^{K - 1} {{X_e}({{\zeta}_r},f)} / K }$. This evokes shifting ${{\cal T}_{r}}$ and the aligning of $w(t)$. We show in the following parts of the paper that ${X_e}({{\zeta}_r},f)$ is capable of delivering reliable spectrum sensing routine provided adequate set-up conditions. This involves selecting $T_{0}$, the average sampling rate $\alpha = {N / {T_0} }$ and $K$.

### A. Evaluation of the Estimator's Adequacy

In order to determine the appropriateness of (5) to the detection purpose, the expected value of the estimator is scrutinised. Since ${\left\{{t_n} \right\}_{n = 1}^N}$ in (5) are i.i.d. random variables, we can write

$$\eqalignno{E &\left[{\left. {{X_{e}}({{\zeta}_{r}},f)} \right\vert x(t)} \right] \cr =&\,{{{T_{0}^{2}}} \over {N(N - 1)\mu}} \left\{NE\left[{\left. {{y^{2}}({t_{n}}) {w^{2}}({t_{n}})} \right\vert x(t)} \right] \right. \cr &+ \left. N(N - 1)E\left[\left.{y({t_{n}})w({t_{n}}){e^{- j2\pi f{t_{n}}}}} \right\vert x(t) \right] \right. \cr &\times \left. E\left[\left. {y({t_{n}})w({t_{n}}){e^{j2\pi f{t_{n}}}}} \right\vert x(t) \right] \right\} &{\hbox{(6)}}}$$

and then

$$\displaylines{E\left[{\left. {{X_{e}}({{\zeta}_{r}},f)} \right\vert x(t)} \right] = {N \over {(N - 1)\mu \alpha}} \hfill\cr\hfill \times \left[{\int_{{{\zeta}_{r}}}^{{{\zeta}_{r}} + {T_{0}}} {{x^{2}}(t){w^{2}}(t)dt} + \mu {\sigma _{n}^{2}}} \right] + {1 \over \mu } {\left\vert {{X_{W}}({{\zeta}_{r}},f)} \right\vert^{2}} \quad{\hbox{(7)}}}$$

noting that the signal and AWGN are independent as well as $E\left[{\left. {x({t_n})w({t_n})} \right \vert x(t)} \right] = \int_{{{\cal T}_r}} {x{{(t)w(t)dt} / {T_0}}}$. Equation (7) emerges as

$$\eqalignno{C({{\zeta}_{r}},f) =&\, E\left[{{X_{e}}({{\zeta}_{r}},f)} \right] \cr = &\, {{N\left[{{P_{S}}({{\zeta}_{r}}) + {\sigma _{n}^{2}}} \right]} \over {(N - 1)\alpha}} + {1 \over \mu }E\left[{{{\left\vert {{X_{W}}({{\zeta}_{r}},f)} \right\vert}^{2}}} \right] . &{\hbox{(8)}}}$$

The signal's weighted power within ${\cal T}_{r}$ is

$${P_{S}}({{\zeta}_{r}}) = {1 \over \mu }\int_{{{\zeta}_{r}}}^{{{\zeta}_{r}} + {T_{0}}} {E\left[{{x^{2}}(t)} \right]{w^{2}}(t)dt} \eqno{\hbox{(9)}}$$

and ${X_W}({{\zeta}_r},f) = \int_{{{\zeta}_r}}^{{{\zeta}_r} + {T_0}} {x(t)w(t){e^{- j2\pi ft}}dt}$ is the windowed Fourier transform of signal $x(t)$ in (1).

It can be noticed from (8) that $C({{\zeta}_r},f)$ consists of a constant frequency-independent component and the expected value of a continuous-time periodogram, i.e., ${{E\left[{{{\left\vert {{X_W}({{\zeta}_r},f)} \right\vert}^2}} \right]} / \mu }$. The former is commonly referred to as smeared-aliasing and is owed to utilizing nonuniform sampling [14]. It is a white-noise-like component existing at all frequencies that would not overshadow any distinctive features of ${{E\left[{{{\left\vert {{X_W}({{\zeta}_r},f)} \right\vert}^2}} \right]} / \mu }$ related to an active transmission. Below, we show that $E\left[{{{\left\vert {{X_W}({{\zeta}_r},f)} \right\vert}^2}} \right]$ serves as a detectable spectral component for the $M$ active subbands and is independent of ${{\zeta}_r}$ at certain frequencies. First, we can write

$$\eqalignno{& E\left[{{{\left\vert {{X_{W}}({{\zeta}_{r}},f)} \right\vert}^{2}}} \right] \cr & = \sum\limits_{m = 1}^{M} {\int_{-\infty }^{+ \infty}}\! \!{w(t)\int_{- \infty }^{+ \infty }\! {{R_{X,m}}(t,t + \tau)w(t + \tau){e^{- j2\pi f\tau}}d\tau dt} } . \cr&&{\hbox{(10)}}}$$

Define: ${H_m}(f) = \int_{- \infty }^{+ \infty } {{h_m}(t){e^{- j2\pi ft}}dt}$, ${P_{i,m}}(f) = \int_{- \infty }^{+ \infty } {{p_{i,m}}(t){e^{- j2\pi ft}}dt}$ and ${P_{q,m}}(f) = \int_{- \infty }^{+ \infty } {{p_{q,m}}(t){e^{- j2\pi ft}}dt}$. For the simplicity of the notation let: ${{\mathop{P\kern0pt}\limits^{\frown}}_{i,m}}(f) = {H_m}(f + {f_{C,m}}){P_{i,m}}(f)$ and ${{\mathop{P\kern0pt}\limits^{\smile}}_{i,m}}(f) = {H_m}(f - {f_{C,m}}){P_{i,m}}(f)$ whereas ${{\mathop{P\kern0pt}\limits^{\frown}}_{q,m}}(f) = {H_m}(f + {f_{C,m}}){P_{q,m}}(f)$ and ${{\mathop{P\kern0pt}\limits^{\smile}}_{q,m}}(f) = {H_m}(f - {f_{C,m}}){P_{q,m}}(f)$. The FT of ${R_{X,m}}(t,t + \tau)$ with respect to the time difference $\tau$ in (10) can be shown to reduce to (11), shown at the bottom of the page

$$\eqalignno{{\cal{F}} \left\{{{R_{X,m}}(t,t + \tau),\tau } \right\} &= 0.25{f_{S,m}}{\sigma _{a,m}^{2}}{e^{- j2\pi n{f_{S,m}}t}} \cr &\times \sum\limits_{n = - \infty }^{+ \infty } \Big [{{{\mathop{P\kern0pt}\limits^{\frown}}}_{i,m}}(f - {f_{C,m}}) {{{\mathop{P\kern0pt}\limits^{\frown}}}_{i,m}^{\ast}}(f - {f_{C,m}} + n{f_{S,m}}) \cr &\quad + {{{\mathop{P\kern0pt}\limits^{\smile}} }_{i,m}}(f + {f_{C,m}}){{{\mathop{P\kern0pt}\limits^{\smile}}}_{i,m}^{\ast}}(f + {f_{C,m}} + n{f_{S,m}}) \Big] \cr &+ 0.25{f_{S,m}}{\sigma _{b,m}^{2}}{e^{- j2\pi n{f_{S,m}}t}} \cr & \times \sum\limits_{n = - \infty }^{+ \infty } \Big [{{{\mathop{P\kern0pt}\limits^{\frown}}}_{q,m}}(f - {f_{C,m}}){{{\mathop{P\kern0pt}\limits^{\frown}}}_{q,m}^{\ast}}(f - {f_{C,m}} + n{f_{S,m}}) \cr &+ {{{\mathop{P\kern0pt}\limits^{\smile}} }_{q,m}}(f + {f_{C,m}}) {{{\mathop{P\kern0pt}\limits^{\smile}} }_{q,m}^{\ast}}(f + {f_{C,m}} + n{f_{S,m}})\Big] &{\hbox{(11)}}}$$

noting the bandpass nature of the propagation channel response over the $m$th active subband and assuming ${f_{C,m}} \gg {B_{C}}$ (${X^{\ast}}$ denotes the conjugate of a complex variable $X$); (10) can be restated as

$$\eqalignno{& E\left[{{{\left\vert {{X_{W}}({{\zeta}_{r}},f)} \right\vert}^{2}}} \right] \cr &= \sum\limits_{m = 1}^{M} \int_{- \infty }^{+ \infty }\! w(t)\left[{{\cal{F}} \left\{{{R_{X,m}}(t,t + \tau),\tau } \right\}} \right] \ast \left[{W(f){e^{j2\pi ft}}} \right]dt \cr&&{\hbox{(12)}}}$$

where $W(f) = \int_{{{\zeta}_{r}}}^{{{\zeta}_{r}} + {T_{0}}} {w(t){e^{- j2\pi ft}}dt}$. Substituting (11) into (12) leads to

$$\displaylines{E\left[{{{\left\vert {{X_{W}}({{\zeta}_{r}},f)} \right\vert}^{2}}} \right] \hfill\cr\hfill =\! 0.25\! \sum\limits_{m = 1}^{M} \! {\sigma_{a,m}^{2}}{f_{S,m}}{F_{i,m}}({{\zeta}_{r}},f) \!+\! {\sigma_{b,m}^{2}}{f_{S,m}}{F_{q,m}}({{\zeta}_{r}},f) \quad{\hbox{(13)}}}$$

where

$$\eqalignno{&{F_{i,m}} ({{\zeta}_{r}},f) \cr&\quad= \sum\limits_{n = - \infty }^{+ \infty }\biggl[{{{\mathop{P\kern0pt}\limits^{\frown}}}_{i,m}}(f - {f_{C,m}}){{{\mathop{P\kern0pt}\limits^{\frown}}}_{i,m}^{\ast}}(f - {f_{C,m}} + n{f_{S,m}}) \cr&\qquad\quad+ {{{\mathop{P\kern0pt}\limits^{\smile}} }_{i,m}}(f + {f_{C,m}}){{{\mathop{P\kern0pt}\limits^{\smile}}}_{i,m}^{\ast}}(f + {f_{C,m}} + n{f_{S,m}}) \biggr] \cr&\qquad\ast \left[{W(f){W^{\ast}}(f - n{f_{S,m}})} \right] &{\hbox{(14)}}}$$

$$\eqalignno{&{F_{q,m}} ({{\zeta}_{r}},f) \cr&\quad= \sum\limits_{n = - \infty }^{+ \infty } \biggl[{{{\mathop{P\kern0pt}\limits^{\frown}}}_{q,m}}(f - {f_{C,m}}){{{\mathop{P\kern0pt}\limits^{\frown}}}_{q,m}^{\ast}}(f - {f_{C,m}} + n{f_{S,m}}) \cr&\qquad\quad+ {{{\mathop{P\kern0pt}\limits^{\smile}}}_{q,m}}(f + {f_{C,m}}) {{{\mathop{P\kern0pt}\limits^{\smile}}}_{q,m}^{\ast}}(f + {f_{C,m}} + n{f_{S,m}}) \biggr] \cr&\qquad\ast \left[{W(f){W^{\ast}}(f - n{f_{S,m}})} \right] .&{\hbox{(15)}}}$$

However, the baud rate is normally related to the bandwidth ${B_{W,m}}$ of the baseband shaping filters ${p_{i,m}}(t)$ and ${p_{q,m}}(t)$. It is typically limited by

$$0.5{B_{W,m}} < {f_{S,m}} \le {B_{W,m}} \eqno{\hbox{(16)}}$$

where ${B_{W,m}} \le {B_{C}}$ [26]. This implies that

$${P_{i,m}}(f){P_{i,m}}(f + n{f_{S,m}}) = 0$$

and

$${P_{q,m}}(f){P_{q,m}}(f + n{f_{S,m}}) = 0$$

if $n \notin \left\{{- 1,0,1} \right\}$. Employing (14) and (15), (13) simplifies to

$$\eqalignno{& E\left[{{\left\vert {{X_{W}}({{\zeta}_{r}},f)} \right\vert}^{2}} \right] = 0.25\!\sum\limits_{m = 1}^{M} \!{\sigma_{a,m}^{2}}{f_{S,m}}\biggl\{\biggl[{{\left\vert {{{{\mathop{P\kern0pt}\limits^{\frown}}}_{i,m}}(f - {f_{C,m}})} \right\vert}^{2}} \cr&+ {{\left\vert {{{{\mathop{P\kern0pt}\limits^{\smile}} }_{i,m}}(f + {f_{C,m}})} \right\vert}^{2}} \biggr] \ast {{\left\vert {W(f)} \right\vert}^{2}} + {\varepsilon_{i,m}}({{\zeta}_{r}},f) \biggr\} \cr &+ {\sigma_{b,m}^{2}}{f_{S,m}}\biggl\{\left[{{{\left\vert {{{{\mathop{P\kern0pt}\limits^{\frown}}}_{q,m}}(f - {f_{C,m}})} \right\vert}^{2}} + {{\left\vert {{{{\mathop{P\kern0pt}\limits^{\smile}}}_{q,m}}(f + {f_{C,m}})} \right\vert}^{2}}} \right]\cr& \ast {{\left\vert {W(f)} \right\vert}^{2}} + {\varepsilon_{q,m}}({{\zeta}_{r}},f) \biggr\} &{\hbox{(17)}}}$$

such that ${\varepsilon_{i,m}}({{\zeta}_{r}},f)$ and ${\varepsilon_{q,m}}({{\zeta}_{r}},f)$ are the components of the summation in (14) and (15), respectively, when $n = \pm 1$. Hence $E\left[{{{\left\vert {{X_{W}}({{\zeta}_{r}},f)} \right\vert}^{2}}} \right]$ embodies distinctive distinguishable features depicted by the tapered squared magnitude of the Fourier transform of the transmission filters shaped by the propagation channel response. According to (14) and (15), ${\varepsilon_{i,m}}({{\zeta}_{r}},f)$ and ${\varepsilon_{q,m}}({{\zeta}_{r}},f)$ are of zero values at the center of the active system subband provided that (16) is satisfied. This affirms that $E\left[{{{\left\vert {{X_{W}}({{\zeta}_{r}},f)} \right\vert}^{2}}} \right]$ at the central part of an active transmission subband, referred to thereafter as the guarded region, is independent of the position of the time analysis window and poses as the detectable feature in $C({{\zeta}_{r}},f)$.

Therefore, the adopted estimator is an admissible tool to unveil the presence of an active transmission where the examined frequency points ${\left\{{{f_{k}}} \right\}_{k = 1}^{L}}$ in (2) are placed at/near the center of the system subbands, i.e., within the identified guarded regions. It is noted that for WSS signals, the used estimator is a suitable tool for detection where $E\left[{{{\left\vert {{X_{W}}({{\zeta}_{r}},f)} \right\vert}^{2}}} \right]$ is independent of ${{\zeta}_{r}}$ for all $f$ [24].

### B. Estimator's Accuracy

The estimator ${X_{e}}({{\zeta}_{r}},f)$ can be reliably used for spectrum sensing only if the difference $\Lambda ({{\zeta}_{r}},f) = \left\vert {C({{\zeta}_{r}},f) - {X_{e}}({{\zeta}_{r}},f)} \right\vert$ is relatively small for a single realization of $x(t)$, especially at the frequency points ${\left\{{{f_{k}}} \right\}_{k = 1}^{L}}$ in (2). Chebychev's inequality states that $\Lambda ({{\zeta}_{r}},f)$ is directly related to the standard deviation of the estimator, i.e., $\Pr \left\{{\left\vert {X - \bar X} \right\vert \ge \kappa {\sigma_{X}}} \right\} \le {1/ {\kappa ^{2}}}$ where $X$ is a random variable, $\bar X = E\left[X \right]$, ${\sigma_{X}^{2}}$ is the variable's variance and $\kappa >0$ [28]. The variance of $X_{e}({\zeta}_{r},f)$, i.e.,

$$\eqalignno{&{\sigma_{e}^{2}}({{\zeta}_{r}},f) \cr & = {\left\{{{N \over {(N - 1)\mu}}} \right\}^{2}}{\rm var} \left\{{{{\left\vert {{{{T_{0}}} \over N}\sum\limits_{n = 1}^{N} {y({t_{n}})w({t_{n}}){e^{- j2\pi f{t_{n}}}}} } \right\vert}^{2}}} \right\} \cr&&{\hbox{(18)}}}$$

should be evaluated in order to ensure the dependability of the SARS method. First

$$\eqalignno{{\left\vert {{X_{TRS}}({{\zeta}_{r}},f)} \right\vert^{2}} =&\, {\left\vert {{{{T_{0}}} \over N}\sum\limits_{n = 1}^{N} {y({t_{n}})w({t_{n}}){e^{- j2\pi f{t_{n}}}}} } \right\vert^{2}} \cr =&\, {R_{TRS}^{2}}({{\zeta}_{r}},f) + {I_{TRS}^{2}}({{\zeta}_{r}},f) &{\hbox{(19)}}}$$

such that

$$\eqalignno{{R_{TRS}}({{\zeta}_{r}},f) =&\, {{{T_{0}}} \over N}\sum\limits_{n = 1}^{N} {y({t_{n}})w({t_{n}})\cos \left({2\pi f{t_{n}} - \theta ({{\zeta}_{r}},f)} \right)} \cr &&{\hbox{(20)}}\cr {I_{TRS}}({{\zeta}_{r}},f) =&\, {{{T_{0}}} \over N}\sum\limits_{n = 1}^{N} {y({t_{n}})w({t_{n}})\sin \left({2\pi f{t_{n}} - \theta ({{\zeta}_{r}},f)} \right)} .\cr &&{\hbox{(21)}}}$$

The phase-shift $\theta ({{\zeta}_{r}},f)$ is chosen in a way that

$${\mathtilde R_{TRS}}({{\zeta}_{r}},f) = {{T_{0}}\sum _{n = 1}^{N} {y({t_{n}})w({t_{n}})\cos \left({2\pi f{t_{n}}} \right)} / N}$$

and

$${\mathtilde I_{TRS}}({{\zeta}_{r}},f) = {{T_{0}}\sum _{n = 1}^{N} {y({t_{n}})w({t_{n}})\sin \left({2\pi f{t_{n}}} \right)} / N}$$

are uncorrelated, i.e., $c({{\zeta}_{r}},f) = E [{{{\mathtilde R}_{TRS}}({{\zeta}_{r}},f){{\mathtilde I}_{TRS}}({{\zeta}_{r}},f)}]$ $= 0$. It does not alter the definition of the estimator in (5) since ${\left\vert {{X_{TRS}}({{\zeta}_{r}},f){e^{j\theta ({{\zeta}_{r}},f)}}} \right\vert^{2}} = {\left\vert {{X_{TRS}}({{\zeta}_{r}},f)} \right\vert^{2}}$. Each of ${R_{TRS}}({{\zeta}_{r}},f)$ and ${I_{TRS}}({{\zeta}_{r}},f)$ is the sum of $N$ independent random variables, thus according to the central limit theorem they can be assumed to be approximately normally distributed for large $N$. In practice, moderate values of $N$ suffice for such an approximation [3]. As a result, ${\left\vert {{X_{TRS}}({{\zeta}_{r}},f)} \right\vert^{2}}$ has approximately an unnormalised chi-squared distribution with two degrees of freedom [28] and the estimator ${X_{e}}({{\zeta}_{r}},f)$ variance is defined by

$${\sigma_{e}^{2}}({{\zeta}_{r}},f) = 2{\left\{{{N \over {(N - 1)\mu}}} \right\}^{2}}\left[{{\sigma_{{R_{TRS}}}^{4}}({{\zeta}_{r}},f) + {\sigma_{{I_{TRS}}}^{4}}({{\zeta}_{r}},f)} \right] \eqno{\hbox{(22)}}$$

where

$$\eqalignno{{\sigma_{{R_{TRS}}}^{2}}({{\zeta}_{r}},f) =&\, {{{\lambda_{C}}({{\zeta}_{r}},f)} \over \alpha } + {{N - 1} \over N}E\left[{{R_{W}^{2}}({{\zeta}_{r}},f)} \right] &{\hbox{(23)}}\cr {\sigma_{{I_{TRS}}}^{2}}({{\zeta}_{r}},f) =&\, {{{\lambda_{S}}({{\zeta}_{r}},f)} \over \alpha } + {{N - 1} \over N}E\left[{{I_{W}^{2}}({{\zeta}_{r}},f)} \right] &{\hbox{(24)}}\cr {\lambda_{C}}({{\zeta}_{r}},f) =&\, \int_{{{\zeta}_{r}}}^{{{\zeta}_{r}} + {T_{0}}} \left\{{E\left[{{x^{2}}(t)} \right] + {\sigma_{n}^{2}}} \right\}{w^{2}}(t) \cr&\times {{\cos }^{2}}\left({2\pi ft - \theta ({{\zeta}_{r}},f)} \right)dt &{\hbox{(25)}}\cr E\left[{{R_{W}^{2}}({{\zeta}_{r}},f)} \right] =&\, \int_{{{\zeta}_{r}}}^{{{\zeta}_{r}} + {T_{0}}} \int_{{{\zeta}_{r}}}^{{{\zeta}_{r}} + {T_{0}}} {{R_{X}}({t_{1}},{t_{2}})} w({t_{1}})w({t_{2}}) \cr&\times \cos \left({2\pi f{t_{1}} - \theta ({{\zeta}_{r}},f)} \right) \cr&\times \cos \left({2\pi f{t_{2}} - \theta ({{\zeta}_{r}},f)} \right)d{t_{1}}d{t_{2}} &{\hbox{(26)}} \cr {\lambda_{S}}({{\zeta}_{r}},f) =&\, \int_{{{\zeta}_{r}}}^{{{\zeta}_{r}} + {T_{0}}} \left\{{E\left[{{x^{2}}(t)} \right] + {\sigma_{n}^{2}}} \right\}{w^{2}}(t) \cr&\times {{\sin }^{2}}\left({2\pi ft - \theta ({{\zeta}_{r}},f)} \right)dt &{\hbox{(27)}}\cr\cr E\left[{{I_{W}^{2}}({{\zeta}_{r}},f)} \right] =&\, \int_{{{\zeta}_{r}}}^{{{\zeta}_{r}} + {T_{0}}} \int_{{{\zeta}_{r}}}^{{{\zeta}_{r}} + {T_{0}}} {{R_{X}}({t_{1}},{t_{2}})} w({t_{1}})w({t_{2}}) \cr&\times \sin \left({2\pi f{t_{1}} - \theta ({{\zeta}_{r}},f)} \right) \cr&\times \sin \left({2\pi f{t_{2}} - \theta ({{\zeta}_{r}},f)} \right)d{t_{1}}d{t_{2}} .&{\hbox{(28)}}}$$

The phase-shift in (20)–(28), whose role is to simplify the estimator's variance expression, is given by

$$\theta ({{\zeta}_{r}},f) \!=\! 0.5{\rm arccot}\left({{{E\left[{{{\mathtilde R}_{TRS}^{2}}({{\zeta}_{r}},f)} \right] \!-\! E\left[{{{\mathtilde I}_{TRS}^{2}}({{\zeta}_{r}},f)} \right]} \over {2c({{\zeta}_{r}},f)}}} \right) \eqno{\hbox{(29)}}$$

where $E\left[{{{\mathtilde R}_{TRS}^{2}}({{\zeta}_{r}},f)} \right]$ and $E\left[{{{\mathtilde I}_{TRS}^{2}}({{\zeta}_{r}},f)} \right]$ are identical to (23) and (24), respectively, such that $\theta ({{\zeta}_{r}},f)$ is discarded from all the terms in (25)–(28). Whereas

$$c({{\zeta}_{r}},f) = {{{\lambda_{CS}}({{\zeta}_{r}},f)} \over \alpha } + {{N - 1} \over N}E\left[{{R_{W}}({{\zeta}_{r}},f){I_{W}}({{\zeta}_{r}},f)} \right] \eqno{\hbox{(30)}}$$

such that

$$\eqalignno{{\lambda_{CS}} ({{\zeta}_{r}},f) =&\, \int_{{{\zeta}_{r}}}^{{{\zeta}_{r}} + {T_{0}}} \left\{{E\left[{{x^{2}}(t)} \right] + {\sigma_{n}^{2}}} \right\}{w^{2}}(t) \cr&\times \cos (2\pi ft)\sin (2\pi ft)dt &{\hbox{(31)}} \cr\cr E \left[{{R_{W}}({{\zeta}_{r}},f){I_{W}}({{\zeta}_{r}},f)} \right] \! =&\, \int_{{{\zeta}_{r}}}^{{{\zeta}_{r}}\! + {T_{0}}} \mkern-12mu \int_{{{\zeta}_{r}}}^{{{\zeta}_{r}}\! + {T_{0}}} \mkern-15mu{R_{X}}\!({t_{1}},{t_{2}})w({t_{1}})w({t_{2}}) \cr&\times \cos (2\pi f{t_{1}})\sin (2\pi f{t_{2}})d{t_{1}}d{t_{2}}. \cr&&{\hbox{(32)}}}$$

Equations (23)–(32) were derived in a similar manner to that of the WSS signals in [24].

The above variance analysis is solely manipulated in establishing reliable spectrum sensing where the proposed SARS method only involves calculating ${X_{e}}({{\zeta}_{r}},f)$. Here, we derive a simplified approximation of the variance's expression in (22)–(32) for the set of assessed frequency points ${f_{k}} \in \left[{{f_{1}},{f_{2}}, \ldots {f_{L}}} \right]$ according to the detection criterion in (2), i.e., one per monitored subband. From (25)

$$\displaylines{{\lambda_{C}}({{\zeta}_{r}},{f_{k}}) = 0.5\int_{{{\zeta}_{r}}}^{{{\zeta}_{r}} + {T_{0}}} \left\{{E\left[{{x^{2}}(t)} \right] + {\sigma_{n}^{2}}} \right\}{w^{2}}(t)\hfill\cr\hfill\times\left\{{1 + \cos \left({4\pi {f_{k}}t - 2\theta ({{\zeta}_{r}},{f_{k}})} \right)} \right\}dt \quad{\hbox{(33)}}}$$

where the term that includes the sinusoid represents a windowed Cosine transform of the signal's second moment plus a constant at frequency point $2{f_{k}}$ which is a high frequency outside the overseen frequency range. This is expected to be of a negligible value in comparison to $\int_{{{\zeta}_{r}}}^{{{\zeta}_{r}} + {T_{0}}} {\left\{{E\left[{{x^{2}}(t)} \right] + {\sigma_{n}^{2}}} \right\}{w^{2}}(t)dt}$ and similar argument applies to ${\lambda_{S}}({{\zeta}_{r}},f)$ in (27). Thus ${\lambda_{C}}({{\zeta}_{r}},{f_{k}}) \approx {\lambda_{S}}({{\zeta}_{r}},{f_{k}}) \approx {\mu \left[{{P_{S}}({{\zeta}_{r}}) + {\sigma_{n}^{2}}} \right]/ 2}$. Based on the fact that $E\left[{{R_{W}^{2}}({{\zeta}_{r}},f)} \right] + E\left[{{I_{W}^{2}}({{\zeta}_{r}},f)} \right] = E\left[{{{\left\vert {{X_{W}}({{\zeta}_{r}},f)} \right\vert}^{2}}} \right]$, we can write

$$\eqalignno{0.5{\left\{{E\left[{{{\left\vert {{X_{W}}({{\zeta}_{r}},f)} \right\vert}^{2}}} \right]} \right\}^{2}} \le &\, {\left\{{E\left[{{R_{W}^{2}}({{\zeta}_{r}},f)} \right]} \right\}^{2}} \cr + {\left\{{E\left[{{I_{W}^{2}}({{\zeta}_{r}},f)} \right]} \right\}^{2}} \le &\, {\left\{{E\left[{{{\left\vert {{X_{W}}({{\zeta}_{r}},f)} \right\vert}^{2}}} \right]} \right\}^{2}} . &{\hbox{(34)}}}$$

From (22)–(24) noting (34), the variance can be approximated by

$$\eqalignno{{\sigma_{e}}^{2} ({{\zeta}_{r}},{f_{k}}) \approx&\, {{{N^{2}}} \over {{{(N - 1)}^{2}}}} \left\{{{{{\left[{{P_{S}}({{\zeta}_{r}}) + {\sigma_{n}^{2}}} \right]}^{2}}} \over {{\alpha ^{2}}}} \right.\cr &+ \left. {{2\left({N - 1} \right)\left[{{P_{S}}({{\zeta}_{r}}) + {\sigma_{n}^{2}} } \right]E \left[{{{\left\vert {{X_{W}}({{\zeta}_{r}},{f_{k}})} \right\vert}^{2}} } \right]} \over {N\alpha \mu}} \right. \cr &+ \left. 2\eta ({{\zeta}_{r}},{f_{k}}){{\left({{{N - 1} \over {N\mu}}E\left[{{{\left\vert {{X_{W}}({{\zeta}_{r}},{f_{k}})} \right\vert}^{2}}} \right]} \right)}^{2}} \right\} \cr&&{\hbox{(35)}}}$$

where $0.5 \le \eta ({{\zeta}_{r}},{f_{k}}) \le 1$ since

$$\displaylines{{\left\{{E\left[{{R_{W}^{2}}({{\zeta}_{r}},{f_{k}})} \right]} \right\}^{2}} + {\left\{{E\left[{{I_{W}^{2}}({{\zeta}_{r}},{f_{k}})} \right]} \right\}^{2}} \hfill\cr\hfill = \eta ({{\zeta}_{r}},{f_{k}}){\left\{{E\left[{{{\left\vert {{X_{W}}({{\zeta}_{r}},f)} \right\vert}^{2}}} \right]} \right\}^{2}} }$$

e.g., $\eta ({{\zeta}_{r}},{f_{k}}) \approx 0.5$ if $E\left[{{R_{W}^{2}}({{\zeta}_{r}},{f_{k}})} \right] \approx E\left[{{I_{W}^{2}}({{\zeta}_{r}},{f_{k}})} \right]$. Deciding the value of $\eta ({{\zeta}_{r}},{f_{k}})$ is important as it forms a substantial part of the estimator's variance. We have: $E\left[{{R_{W}^{2}}({{\zeta}_{r}},f)} \right] = {\psi_{1}}({{\zeta}_{r}},f) + {\psi_{2}}({{\zeta}_{r}},f)$ and $E\left[{{I_{W}^{2}}({{\zeta}_{r}},f)} \right] = {\psi_{1}}({{\zeta}_{r}},f) - {\psi_{2}}({{\zeta}_{r}},f)$ such that

$$\eqalignno{{\psi_{1}}({{\zeta}_{r}},f) =&\, 0.5\sum\limits_{m = 1}^{M} \int_{{{\zeta}_{r}}}^{{{\zeta}_{r}} + {T_{0}}} \int_{{{\zeta}_{r}}}^{{{\zeta}_{r}} + {T_{0}}} {R_{X,m}}({t_{1}},{t_{2}})w({t_{1}})w({t_{2}})\cr&\times\cos \left({2\pi f({t_{1}} - {t_{2}})} \right)d{t_{1}}d{t_{2}} &{\hbox{(36)}}\cr {\psi_{2}}({{\zeta}_{r}},f) =&\, 0.25\sum\limits_{m = 1}^{M} {e^{j2\theta ({{\zeta}_{r}},f)}}{G_{m}}({{\zeta}_{r}},f) \cr&+ {e^{- j2\theta ({{\zeta}_{r}},f)}}{G_{m}^{\ast}}({{\zeta}_{r}},f) &{\hbox{(37)}}}$$

where ${G_{m}}({{\zeta}_{r}},f) = {\sigma_{a,m}^{2}}{G_{i,m}}({{\zeta}_{r}},f) + {\sigma_{b,m}^{2}}{G_{q,m}}({{\zeta}_{r}},f)$. Whereas

$$\eqalignno{{G_{i,m}}({{\zeta}_{r}},f) =&\, \sum\limits_{n = - \infty }^{+ \infty } \int_{{{\zeta}_{r}}}^{{{\zeta}_{r}} + {T_{0}}} \int_{{{\zeta}_{r}}}^{{{\zeta}_{r}} + {T_{0}}} {{s_{i,m}}({t_{1}},n){s_{i,m}}({t_{2}},n)} \cr&\times w({t_{1}})w({t_{2}}){e^{- j2\pi f({t_{1}} + {t_{2}})}}d{t_{1}}d{t_{2}} &{\hbox{(38)}}\cr {G_{q,m}}({{\zeta}_{r}},f) =&\, \sum\limits_{n = - \infty }^{+ \infty } \int_{{{\zeta}_{r}}}^{{{\zeta}_{r}} + {T_{0}}} \int_{{{\zeta}_{r}}}^{{{\zeta}_{r}} + {T_{0}}} {s_{q,m}}({t_{1}},n){s_{q,m}}({t_{2}},n)\cr&\times w({t_{1}})w({t_{2}}){e^{- j2\pi f({t_{1}} + {t_{2}})}}d{t_{1}}d{t_{2}} .&{\hbox{(39)}}}$$

Hence ${\psi_{2}}({{\zeta}_{r}},f)$ sets the equivalent $\eta ({{\zeta}_{r}},f)$ value in (35) given (34) where $E\left[{{R_{W}^{2}}({{\zeta}_{r}},f)} \right] - E\left[{{I_{W}^{2}}({{\zeta}_{r}},f)} \right] = 2{\psi_{2}}({{\zeta}_{r}},f)$; $\eta ({{\zeta}_{r}},f) \approx 0.5$ only if ${\psi_{2}}({{\zeta}_{r}},f) \approx 0$ which is the case for WSS signals [24]. To depict the impact of cyclostationarity on (35), we assume that the FT of the $w(t)$ reduces to a Dirac delta $\delta (f)$, i.e., very long time analysis window. Each of (38) and (39) emerges as

$$\eqalignno{G &_{i,m} ({{\zeta}_{r}},f) \cr =&\, 0.25{\left[{{H_{m}}(f)} \right]^{2}}\sum\limits_{n = - \infty }^{+ \infty } {{\left[{{P_{i,m}}(f - {f_{C,m}})} \right]}^{2}}{e^{j4\pi (f - {f_{C,m}})n{T_{S,m}}}} \cr &+ {{\left[{{P_{i,m}}(f + {f_{C,m}})} \right]}^{2}}{e^{j4\pi (f + {f_{C,m}})n{T_{S,m}}} } &{\hbox{(40)}} \cr\cr G&_{q,m}({{\zeta}_{r}},f) \cr \!=&\,\! -\! 0.25{\left[{{H_{m}}(f)} \right]^{2}}\!\!\sum\limits_{n = - \infty }^{+ \infty } \!\! {{\left[{{P_{q,m}}(f \!-\! {f_{C,m}})} \right]}^{2}}{e^{j4\pi (f - {f_{C,m}})n{T_{S,m}}}} \cr &+ {{\left[{{P_{q,m}}(f + {f_{C,m}})} \right]}^{2}}{e^{j4\pi (f + {f_{C,m}})n{T_{S,m}}}} &{\hbox{(41)}}}$$

upon taking the Fourier transforms in (38) and (39) with respect to ${t_{1}}$ and ${t_{2}}$ separately. Thus we obtain

$$\eqalignno{{G_{m}} & ({{\zeta}_{r}},f) \cr =&\, 0.125{f_{S,m}} {\left[{{H_{m}}(f)} \right]^{2}} \cr &\times \left\{{{\sigma_{a,m}^{2}}{{\left[{{P_{i,m}}(f - {f_{C,m}})} \right]}^{2}} - {\sigma_{b,m}^{2}}{{\left[{{P_{q,m}}(f - {f_{C,m}})} \right]}^{2}}} \right\} \cr &\times \sum\limits_{n = - \infty }^{+ \infty } {\delta (f - {f_{C,m}} - 0.5n{f_{S,m}})} \cr &+ 0.125{f_{S,m}}{\left[{{H_{m}}(f)} \right]^{2}}\left\{{\sigma_{a,m}^{2}}{{\left[{{P_{i,m}}(f + {f_{C,m}})} \right]}^{2}} \right. \cr &- \left.{\sigma_{b,m}^{2}}{{\left[{{P_{q,m}}(f \!+\! {f_{C,m}})} \right]}^{2}} \right\} \!\!\sum\limits_{n = - \infty }^{+ \infty } {\delta (f \!+\! {f_{C,m}} \!-\! 0.5n{f_{S,m}})} \cr &&{\hbox{(42)}}}$$

by utilizing Fourier expansion. Equation (42) shows that ${\psi_{2}}({{\zeta}_{r}},f)$ in (37) can have nonzero values concentrated at frequencies equal to shifted multiples of half of the symbol rate, i.e., $\pm {f_{C,m}} - 0.5n{f_{S,m}}$ ($n \in \BBZ$), and belong to the $m$th active subband provided that ${\sigma_{a,m}^{2}}{P_{i,m}}(f) \ne {\sigma_{b,m}^{2}}{P_{q,m}}(f)$. For a range of modulations schemes, e.g., quadrature amplitude modulation (QAM) and quadrature phase shift keying (QPSK), this condition is not satisfied since ${\sigma_{a,m}^{2}} = {\sigma_{b,m}^{2}}$ and identical shaping filters are commonly used in the in-phase and quadrature branches, i.e., ${\left[{{P_{i,m}}(f)} \right]^{2}} - {\left[{{P_{q,m}}(f)} \right]^{2}} = 0$. Clearly, in this case ${\psi_{2}}({{\zeta}_{r}},f) = 0$ and $\eta ({{\zeta}_{r}},f) = 0.5$ is commensurate within the $m$th active subband. Any mismatch between these two branches, i.e., ${\sigma_{a,m}^{2}}{P_{i,m}}(f) - {\sigma_{b,m}^{2}}{P_{q,m}}(f) \ne 0$, can lead to discrepancies between $E\left[{{R_{W}^{2}}({{\zeta}_{r}},f)} \right]$ and $E\left[{{I_{W}^{2}}({{\zeta}_{r}},f)} \right]$ within the corresponding transmission subband. This can result in surges in the variance values at selected frequency points according to (34), (37), and (42). For a binary phase shift keying (BPSK) signal where only an in-phase component is present, (42) becomes

$$\eqalignno{{G_{m}}&({{\zeta}_{r}},f) =\, 0.125{f_{S,m}}{\sigma_{a,m}^{2}}{\left[{{H_{m}}(f)} \right]^{2}} \cr &\times \sum\limits_{n = - \infty }^{+ \infty } {{\left[{{P_{i,m}}(f - {f_{C,m}})} \right]}^{2}}\delta (f - {f_{C,m}} - 0.5n{f_{S,m}}) \cr &+ {{\left[{{P_{i,m}}(f + {f_{C,m}}) } \right]}^{2}}\delta (f + {f_{C,m}} - 0.5n{f_{S,m}}) .}$$

This indicates that ${\psi_{2}}({{\zeta}_{r}},f)$ and consequently $\eta ({{\zeta}_{r}},f)$ can tend to their maximum values producing a notable deterioration in the estimator's accuracy at frequencies ${f_{n}} = \pm {f_{C,m}} - 0.5n{f_{S,m}}$, $n \in \BBZ$, such that ${f_{n}}$'s belong to the subband's frequency range ${B_{W,m}}$. This is the case for any other linear modulation scheme that has only one branch, i.e., either in-phase or quadrature. Therefore, the accuracy of the spectrum estimator can be affected by the signal's cyclostationarity and any processing task that relies on the spectral analysis, e.g., spectrum sensing, should consider the possible presence of such phenomenon. In the following subsection, we give a numerical example to illustrate the estimator's response to processing two types of cyclostationary signals.

### C. Numerical Example of the Estimator's Variance

**Fig. 1.**
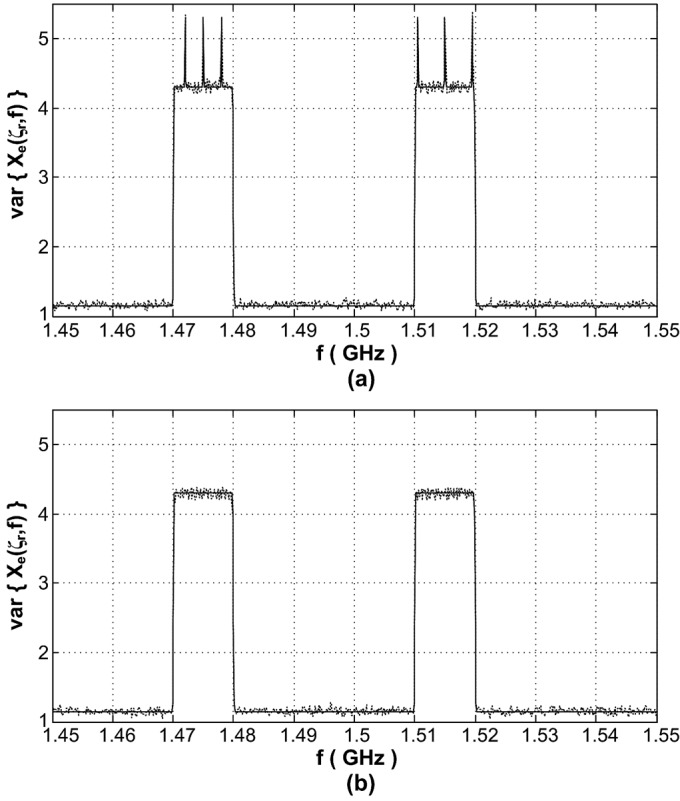
Estimator's variance from equations (solid line) and MSE (dotted line). (a) For BPSK modulation. (b) For 16QAM modulation.

Consider a multiband communication system comprising 10 subbands occupying the frequency range [1.45, 1.55] GHz, i.e., ${B_{C}} = 10 {\rm MHz}$. A Blackman window of width ${T_{0}} = 10 \mu {\rm s}$ and an average sampling rate of $\alpha = 90 {\rm MHz}$ are used. Two of the system subbands are active with similar power levels and the SNR is $- {1.5} {\rm dB}$. Two examples are shown here; in the first one BPSK signals are transmitted whereas in the second example 16QAM modulated signals are conveyed over the active system subbands. In both cases, the symbol rate of the active subband with the central frequency ${f_{{C_{3}}}} = 1.475 {\rm GHz}$ is ${f_{{S_{3}}}} = 6 {{\rm MSym} / {\rm s}}$ and the one centered at ${f_{{C_{7}}}} = 1.515 {\rm GHz}$ has a baud rate of ${f_{{S_{7}}}} = 9 {{\rm MSym} / {\rm s}}$. Fig. 1(a) and (b) show the variance given by (35) and the mean squared error (MSE) obtained from 10000 independent experiments for the BPSK and 16QAM cases, respectively. The corresponding $\eta ({{\zeta}_{r}},f)$ is calculated from (26), (28), and (34).

It can be seen from Fig. 1(a) that the estimator's variance for the BPSK signals grows noticeably at certain frequencies within each of the two active subbands. Those sudden increases take place at/near frequencies ${f_{{n_{3}}}} = {f_{C,3}} - 0.5n{f_{S,3}}$ and ${f_{{n_{7}}}} = {f_{C,7}} - 0.5n{f_{S,7}}$ where $n = \left\{{- 1,0,1} \right\}$ for the subbands centered at ${f_{{C_{3}}}}$ and ${f_{{C_{7}}}}$, respectively. Whereas, in Fig. 1(b) the estimator retains its consistency within the active subbands where $\eta ({{\zeta}_{r}},f) \approx 0.5$. The close match between the analytical and simulation results in both plots in Fig. 1 confirms the accuracy of the conducted calculations. The possible use of a modulation scheme that can lead to degradation in the estimator's performance (e.g., BPSK) by any of the system transmitters should be taken into account when utilizing SARS.

### D. Signal Analysis Window and Estimate Averages

Achieving the minimum sensing time is a highly desirable feature for any spectrum sensing technique, particularly if sensing is a continuous real-time operation that has to fulfill specific time constraints. Hence we aim to use a short time analysis window. Additionally, attaining low resolution spectrographs via a short ${{\cal T}_{r}}$ facilitates minimizing the number of needed frequency points per system subband to establish any activity within, i.e., save on computations noting that one frequency point per subband is examined in (2). However, ${T_{0}}$ should be long enough so that the spectrum tapering does not overshadow the distinctive features of $C({{\zeta}_{r}},f)$. Taking a number of multiples of the recommendation in [24] where ${T_{0}} \ge {n/ {B_{C}}}$ such that $n \ge 1$ serves as a reasonable guideline on choosing the width of the signal time analysis window.

Inspecting ${\sigma_{e}^{2}}({{\zeta}_{r}},f)$, we notice that it is nearly constant at frequencies where there is no spectral activity and its values decrease upon increasing the average sampling rate $\alpha$. The variance has its highest values at frequencies where the signal is present and a substantial part of this inaccuracy is unaffected by $\alpha$. A classical tactic to minimize the latter error is to resort to averaging a number of ${X_{e}}({{\zeta}_{r}},f)$ estimates from $K$ signal windows. The adopted detector involves averaging $K$ number of the ${X_{e}}({{\zeta}_{r}},f)$ estimates from nonoverlapping signal windows of length ${T_{0}}$ such that

$${\mathhat X_{e}}(f) = {1 \over K}\sum\limits_{r = 0}^{K - 1} {{X_{e}}(r{T_{0}},f)} .\eqno{\hbox{(43)}}$$

The nonoverlapping signal segments are assumed to be uncorrelated in this study. Finding the value of $K$ in (43) is essential to realize a dependable sensing strategy and quantify its constraints.

## IV. Multiband Reliable Spectrum Sensing

The reliability and robustness of the SARS technique is reflected by its ability to meet a sought system behavior that is commonly expressed by the receiver operating characteristics. The ROC of each of the system subbands captures the relation between the probability of false alarm, i.e., ${P_{f,k}} = \Pr \left\{{\left. {{H_{1,k}}} \right\vert{H_{0,k}}} \right\}$, and the probability of detection, i.e., ${P_{d,k}} = \Pr \left\{{\left. {{H_{1,k}}} \right\vert{H_{1,k}}} \right\}$. Those probabilities are interrelated via the decision threshold, i.e., ${\gamma_{k}}$ in (2). The proposed method ought to fulfil set sensing requirements using the rule in (2) for the suitable ${\gamma_{k}}$ values. This can be achieved by restricting the possible perturbations/anomalies in the estimated spectrum ${\mathhat X_{e}}(f)$ through the available means, i.e., the average sampling rate $\alpha = {N/ {T_{0}}}$ and the number of the estimate averages $K$. In the following subsection, we formulate the dependability conditions of SARS where the multiband signal consists of the maximum expected number of concurrently active transmissions.

### A. Reliability Conditions

Distinctive ROC plots, i.e., ${P_{d,k}}$ versus ${P_{f,k}}$ for a ${\gamma_{k}}$ sweep where $k = 1,2 \ldots L$, are attained for every combined $\alpha$ and $K$ values as the latter two dictate the statistical characteristics of ${\mathhat X_{e}}(f)$ in consonance with (8), (22)–(28), and (43). We have two vectors ${{\bf P}_{\bf f}} = {\left[{{P_{f,1}},{P_{f,2}}, \ldots ,{P_{f,L}}} \right]^{T}}$ and ${{\bf P}_{\bf d}} = {\left[{{P_{d,1}},{P_{d,2}}, \ldots ,{P_{d,L}}} \right]^{T}}$ describing the desired multiband detection performance. Due to nonuniform sampling, the estimated spectrum suffers from smeared-aliasing defect present at all frequencies and embodies a form of the signal and noise powers as indicated by (8) and (35). Evidently, the subband with the weakest power level or spectral peak ${\mathhat X_{e}}({f_{k}})$ is most susceptible to the estimator's possible erroneousness as it can be overshadowed by the inaccuracies caused by other active subbands. A practical approach to this problem, which is adopted here, is to target a priority subband and guarantee satisfying its ROC characteristics by choosing the adequate average sampling rate and the number of estimate averages. Aiming to detect a weak or high performance subband would demand more estimate averages and/or higher sampling rate compared to a stronger or lower performance one.

The estimator ${X_{e}}({{\zeta}_{r}},f)$ is approximately of a chi-squared distribution with two degrees of freedom and subsequently ${\mathhat X_{e}}(f)$ is of similar distribution but with $2K$ degrees of freedom. We note that the cumulative distribution function (CDF) of an unnormalized chi-squared random variable with $Z$ degrees of freedom can be closely approximated by a normal CDF for moderately large $K$ [28]. Hence the CDF of ${\mathhat X_{e}}({f_{k}})$ can be assumed to be approximately equal to that of a normal distribution with the same mean and variance. This can be further justified by central limit theorem [28] and its accuracy is verified by the simulations in Section V. Accordingly, the CDF complement function mandates the ROC probabilities for a given threshold such that

$${P_{f,k}} = \Pr \left({\left. {{{\mathhat X}_{e}}({f_{k}}) \ge {\gamma_{k}}} \right\vert{H_{0,k}}} \right) = Q\left({{{{\gamma_{k}} - {m_{0,k}}} \over {{\sigma_{0,k}}}}} \right) \eqno{\hbox{(44)}}$$

and

$${P_{d,k}} = \Pr \left({\left. {{{\mathhat X}_{e}}({f_{k}}) \ge {\gamma_{k}}} \right\vert{H_{1,k}}} \right) = Q\left({{{{\gamma_{k}} - {m_{1,k}}} \over {{\sigma_{1,k}}}}} \right) \eqno{\hbox{(45)}}$$

where $Q\left(z \right)$ is the tail probability of a zero mean unit-variance normal random variable and is a monotonically nonincreasing function. Each of ${m_{0,k}} = E\left[{{{\mathhat X}_{e}}({f_{k}})} \right]$ and ${\sigma_{0,k}} = \sqrt {{\rm var} \left[{{{\mathhat X}_{e}}({f_{k}})} \right]}$ are for ${H_{0,k}}$, i.e., when subband $k$ is inactive whereas ${m_{1,k}} = E\left[{{{\mathhat X}_{e}}({f_{k}})} \right]$ and ${\sigma_{1,k}} = \sqrt {{\rm var} \left[{{{\mathhat X}_{e}}({f_{k}})} \right]}$ are for ${H_{1,k}}$, i.e., when subband $k$ is active.

The objective is to achieve: ${P_{f,k}} \le {\Delta_{k}}$ and ${P_{d,k}} \ge {\ell_{k}}$ for a targeted system subband referred to in the sequel by subscript $k$. As a result, the corresponding threshold values are

$${\gamma_{k}} \ge {m_{0,k}} + {Q^{- 1}}\left({{\Delta_{k}}} \right){\sigma_{0,k}} \eqno{\hbox{(46)}}$$

for ${H_{0,k}}$ where ${P_{f,k}} \le {\Delta_{k}}$ and

$${\gamma_{k}} \le {m_{1,k}} + {Q^{- 1}}\left({{\ell_{k}}} \right){\sigma_{1,k}} \eqno{\hbox{(47)}}$$

for ${H_{1,k}}$ where ${P_{d,k}} \ge {\ell_{k}}$. Following (46) and (47), we can write

$${m_{1,k}} - {m_{0,k}} \ge {Q^{- 1}}\left({{\Delta_{k}}} \right){\sigma_{0,k}} - {Q^{- 1}}\left({{\ell_{k}}} \right){\sigma_{1,k}} .\eqno{\hbox{(48)}}$$

In order to use (48), we have to calculate $\mathtilde C({f_{k}}) = E\left[{{{\mathhat X}_{e}}({f_{k}})} \right]$ and ${\mathtilde \sigma_{e}^{2}}({f_{k}}) = {\rm var} \left[{{{\mathhat X}_{e}}({f_{k}})} \right]$. Given (8), it can be shown that

$$\mathtilde C({f_{k}}) = {N \over {(N - 1)\alpha}}\left[{{P_{SA}} + {P_{N}}} \right] + {1 \over \mu }E\left[{{{\left\vert {{X_{W}}({{\zeta}_{r}},{f_{k}})} \right\vert}^{2}}} \right] \eqno{\hbox{(49)}}$$

noting that $E\left[{{{\left\vert {{X_{W}}({{\zeta}_{r}},{f_{k}})} \right\vert}^{2}}} \right]$ is independent of ${{\zeta}_{r}}$ when the ${f_{k}}$ point is in the recognized guarded region in Section III. The noise power is denoted by ${P_{N}} = {\sigma_{n}^{2}}$ and

$${P_{SA}} = {1 \over K}\sum\limits_{r = 0}^{K - 1} {{P_{S}}(r{T_{0}})} \eqno{\hbox{(50)}}$$

where ${P_{S}}({{\zeta}_{r}})$ is defined in (9). To compute

$${\mathtilde \sigma_{e}^{2}}({f_{k}}) = {1 \over {{K^{2}}}}\sum\limits_{r = 0}^{K - 1} {{\sigma_{e}^{2}}(r{T_{0}},{f_{k}})} \eqno{\hbox{(51)}}$$

we utilize the simplified variance expression in (35). Deciding $\eta ({{\zeta}_{r}},{f_{k}})$ is of paramount importance as it stipulates a substantial part of the variance. If no previous knowledge is available on the employed modulation schemes and the symbol rates of the transmitted messages, a conservative approach to this predicament is to take into account the worst case scenario, i.e., $\mathhat \eta = \eta ({{\zeta}_{r}},{f_{k}}) = 1$. Nonetheless, any prior information about the incoming signal can be used to set $\mathhat \eta$ or possibly choose the position of the frequency points ${\left\{{{f_{k}}} \right\}_{k = 1}^{L}}$ in (2) to avoid any undesired frequencies where the accuracy of the estimation process deteriorates noticeably with the aid of (22)–(28). Substituting the individual ${\sigma_{e}^{2}}({{\zeta}_{r}},{f_{k}})$ into (51), we arrive at

$$\displaylines{{\mathtilde \sigma_{e}^{2}}({f_{k}}) \approx {{{N^{2}}} \over {{{(N - 1)}^{2}}K}} \bigg\{{{{{P^{\prime}}_{\!\!SA}} + 2{P_{SA}}{P_{N}} + {P_{N}^{2}}} \over {{\alpha ^{2}}}} \hfill\cr\hfill + {{2\left({N - 1} \right)\left({{P_{SA}} + {P_{N}}} \right){D_{k}}} \over {N\alpha}} + 2\mathhat \eta {{\left({{{N - 1} \over N}{D_{k}}} \right)}^{2}} \bigg\} \quad{\hbox{(52)}}}$$

where ${D_{k}} = {E\left[{{{\left\vert {{X_{W}}({{\zeta}_{r}},{f_{k}})} \right\vert}^{2}}} \right]/ \mu }$ and

$${P^{\prime}_{SA}} = {1 \over K}\sum\limits_{r = 0}^{K - 1} {{P_{S}^{2}}(r{T_{0}})} .\eqno{\hbox{(53)}}$$

The signal powers in (50) and (53) varies depending on the activity of the targeted subband $k$. The reliability guidelines in practice should cater for severe system conditions. Those include: the ${L_{A}}$ strongest system subbands are simultaneously active when the $k$th subband is idle, i.e., ${L_{A,0}}$ for ${H_{0,k}}$, and the ${L_{A}} - 1$ strongest subbands are concurrently active when the $k$th subband is engaged, i.e., ${L_{A,1}}$ for ${H_{1,k}}$. We indicate each of those powers by “(0)” and “(1)” superscripts to signify ${H_{0,k}}$ and ${H_{1,k}}$, respectively. Thus, in summary, we have

$$\mathtilde C({f_{k}}) = \cases{{{N \over {(N - 1)\alpha}}\left\{{{P_{SA}}^{(0)} + {P_{N}}} \right\}} & for ${{H_{0,k}}}$ \cr {{N \over {(N - 1)\alpha}}\left\{{{P_{SA}}^{(1)} + {P_{N}}} \right\} + {D_{k}}} & for ${{H_{1,k}}}$ }\eqno{\hbox{(54)}}$$

and (55), shown at the bottom of the page.

$${\mathtilde \sigma_{e}^{2}}({f_{k}}) \approx \cases{\hskip8pi{{{{N^{2}}} \over {{{(N - 1)}^{2}}K}}\left\{{{{{{P^{\prime}}_{\!\!SA}}^{(0)} + 2{P_{SA}}^{(0)} {P_{N}} + {P_{N}^{2}}} \over {{\alpha ^{2}}}}} \right\}} & for \ ${{H_{0,k}}}$ \cr {{{N^{2}}} \over {{{(N - 1)}^{2}}K}}\biggl\{{{{{P^{\prime}}_{\!\!SA}}^{(1)} + 2{P_{SA}}^{(1)} {P_{N}} + {P_{N}^{2}}} \over {{\alpha ^{2}}}} + {{2\left({N - 1} \right)\left({{P_{SA}}^{(1)}+ {P_{N}}} \right){D_{k}}} \over {N\alpha}} + 2\mathhat \eta {{\left({{{N - 1} \over N}{D_{k}}} \right)}^{2}} \biggr\} & for \ ${{H_{1,k}}}.$}\eqno{\hbox{(55)}}$$

According to Parseval's theorem

$$\int_{{{\zeta}_{r}}}^{{{\zeta}_{r}} + {T_{0}}} {E\left[{{x^{2}}(t)} \right]{w^{2}}(t)dt = } \int_{- \infty }^{+ \infty } {E\left[{{{\left\vert {{X_{W}}({{\zeta}_{r}},f)} \right\vert}^{2}}} \right]df}$$

hence ${P_{SA}}^{(0)} \le \sum_{n \in {L_{A,0}}} {2{B_{C}}{D_{n}}}$ and ${P_{SA}}^{(1)} \le \sum_{n \in {L_{A,1}}} {2{B_{C}}{D_{n}}}$ approximates the area underneath the integral. Adopting a conservative approach and substituting (54) as well as (55) values into (48), we obtain (56), shown at the bottom of the page,

$$\eqalignno{{{2{B_{C}}N} \over {(N - 1)\alpha}} \left({{\phi_{1,k}} - {\phi_{0,k}}} \right) \!+\! 1 \ge &\, {{2{B_{C}}N{Q^{- 1}}\left({{\Delta_{k}}} \right)} \over {(N - 1)\sqrt K}}\left({{{{\phi_{0,k}} + {\phi_{1,k}}{\rm SNR}^{{- 1}}} \over \alpha}} \right) \cr - {{N{Q^{- 1}}\left({{\ell_{k}}} \right)} \over {(N - 1)\sqrt K}} & \sqrt {\left\{{{{\left[{{{2{B_{C}}{\phi_{1,k}}\left({1 + {\rm SNR}^{{- 1}}} \right)} \over \alpha}} \right]}^{2}} \!+\! {{4{B_{C}}{\phi_{1,k}}(N - 1)\left({1 + {\rm SNR}^{{- 1}}} \right)} \over {N\alpha}} \!+\! 2\mathhat \eta {{\left({{{N - 1} \over N}} \right)}^{2}}} \right\}} &{\hbox{(56)}}}$$

where ${\phi_{0,k}} = \sum_{n \in {L_{A,0}}} {{D_{n}} / {D_{k}}}$ and ${\phi_{1,k}} = \sum_{n \in {L_{A,1}}} {{D_{n}} / {D_{k}}}$ are the ratios of the sum of ${E\left[{{{\left\vert {{X_{W}}({{\zeta}_{r}},{f_{n}})} \right\vert}^{2}}} \right]/ \mu }$ to that of the targeted subband, i.e., ${D_{k}}$. They can be learnt a priori when transmissions are known to be present, e.g., in [2], [3], and [11]. Whereas, ${\rm SNR} = {{P_{SA}}^{(1)} / {P_{N}}}$ is the signal to noise ratio. Following straightforward rendering, (56) emerges as (57), shown at the bottom of the page.

$$K \ge {\left\{{{{{Q^{- 1}}\left({{\Delta_{k}}} \right)\left({{\phi_{0,k}} + {\phi_{1,k}}{\rm SNR}^{{- 1}}} \right) - {Q^{- 1}}\left({{\ell_{k}}} \right)\sqrt {{\phi_{1,k}^{2}}{{\left({1 + {\rm SNR}^{{- 1}}} \right)}^{2}} + {{{\phi_{1,k}}\alpha (N - 1)\left({1 + {\rm SNR}^{{- 1}}} \right)} \over {N{B_{C}}}} + 0.5\mathhat \eta {\alpha ^{2}}{{\left({{{N - 1} \over {N{B_{C}}}}} \right)}^{2}}} } \over {\left({{\phi_{1,k}} - {\phi_{0,k}}} \right) + {0.5(N - 1)\alpha \over N{B_{C}}}}}} \right\}^{2}} \eqno{\hbox{(57)}}$$

Equation (57) gives a conservative lower limit on the number of windows that need to be averaged as a function of the spectrum occupancy, average sampling rate, signal to noise ratio and the sought system performance. This recommendation can be used to decide the required average sampling rate for a number of estimate averages possibly imposed by practical constraints (e.g., latency) in a continuous processing environment. It is a clear indication of the tradeoff between the sampling rate and the number of averages requested in relation to achieving reliable sensing. Equation (57) affirms that the sensing task can be reliably accomplished with arbitrarily low sampling rates at the expense of an infinitely long signal observation window. This confirms early results on DASP, e.g., [21] and [22], which were rather limited to PSD estimation for WSS signals.

The sensing process includes specifying the thresholds in (2), i.e., ${\mmb{\gamma}} = {\left[{{\gamma_{1}},{\gamma_{2}}, \ldots ,{\gamma_{L}}} \right]^{T}}$. By conforming to (46) and (47), we have

$$\displaylines{{{\mmb{\gamma}}_{\bf 0}}^{(\min)} = \left[{m_{0,1}} + {Q^{- 1}}\left({{P_{f,1}}} \right){\sigma_{0,1}},{m_{0,2}} \right. \hfill\cr\hfill +\left. {Q^{- 1}}\left({{P_{f,2}}} \right){\sigma_{0,2}}, \ldots ,{m_{0,L}} + {Q^{- 1}}\left({{P_{f,L}}} \right){\sigma_{0,L}} \right]^{T} \quad{\hbox{(58)}}}$$

and

$$\displaylines{{{\mmb{\gamma}}_{1}}^{(\max)} = \left[{m_{1,1}} + {Q^{- 1}}\left({{P_{d,1}}} \right){\sigma_{1,1}},{m_{1,2}} \right. \hfill\cr\hfill + \left. {Q^{- 1}}\left({{P_{d,2}}} \right){\sigma_{1,2}}, \ldots ,{m_{1,L}} + {Q^{- 1}}\left({{P_{d,L}}} \right){\sigma_{1,L}} \right]^{T} \quad{\hbox{(59)}}}$$

such that

$${{\mmb{\gamma}}_{\bf 0}}^{(\min)} \le {\mmb{\gamma}} \le {{\mmb{\gamma}}_{1}}^{(\max)} \eqno{\hbox{(60)}}$$

where the components of (58) and (59) can be computed according to (54) and (55) for $k = 1,2, \ldots L$. It is noted that correlated or overlapping signal windows scenario can be easily introduced into the SARS technique whenever the effect of correlation/overlapping on the variance reduction following averaging is known, e.g., Welch periodograms [29] and [30].

### B. Randomised Versus Uniform Sampling

Spectrum sensing methods that employ periodogram-type estimators with uniform sampling to detect active transmissions via assessing spectral peak, e.g., [12], typically demand less estimate averaging compared to SARS which suffers from the smeared-aliasing defect. Following similar analysis/methodology to that of the TRS scheme, it can be shown that the number of estimate averages for the uniform-sampling-based algorithm is given by

$$\eqalignno{&{K_{US}} \ge \Bigg\{{{2{B_{C}}{\phi_{_{1,k}}}{\rm SNR}^{{- 1}}{Q^{- 1}}\left({\Delta}_{k} \right)} \over {{f_{US}}}} \cr & - {Q^{- 1}}\left({\ell}_{k} \right)\sqrt {{{4{B_{C}^{2}}{\phi_{1,k}^{2}}{\rm SNR}^{{- 2}}} \over {{f_{US}^{2}}}} + {{4{B_{C}}{\phi_{1,k}}{\rm SNR}^{{- 1}}} \over {{f_{US}}}} + 2\mathhat \eta } \Bigg\}^{2} \cr&&{\hbox{(61)}}}$$

where ${f_{US}}$ is the uniform sampling rate and is proportional to the monitored bandwidth $B$ to avoid the aliasing effects. Comparing the efficiency of both approaches based only on the sampling rates can be regarded as partial. The detection decision in both cases relies on calculating a form of discrete-time Fourier transform from a finite set of the signal samples, e.g., DFT or an optimized version whenever applicable. Therefore, the number of processed samples is a critical factor in deciding the efficiency of the SARS technique and its benefits over the conventional uniform-sampling-based ones. From (57) and (61), the corresponding numbers of processed samples for randomized and uniform sampling approaches are

$${N_{TRS}} \ge {T_{0}}\alpha K \eqno{\hbox{(62)}}$$

and

$${N_{US}} \ge {T_{0}}{f_{US}}{K_{US}} \eqno{\hbox{(63)}}$$

respectively. Generally, the proposed method provides tangible savings not only on the used sampling rate but also on the overall number of processed samples in low spectrum utilization environments, i.e., ${{B_{A}}/ B} \ll 1$. In fact, extending the monitored bandwidth assuming a constant SNR (e.g., the sampling is preceded by a filter to limit the noise bandwidth/power) a fixed number of concurrently active subbands ${B_{A}}$ and same system behavior does not impose any additional cost on the sample numbers for SARS as indicated by (62). On the other hand, the number of requested uniformly distributed samples in such cases grows at a rate equivalent to ${f_{US}}$ where ${f_{US}} \ge 2B$. This shows that as the spectrum occupancy decreases, the benefits of exploiting nonuniform sampling become more visible. Low spectrum utilization is faced in various applications, e.g., in CR networks it can be 15% or lower in certain bands [11].

## V. Numerical Examples

**Fig. 2.**
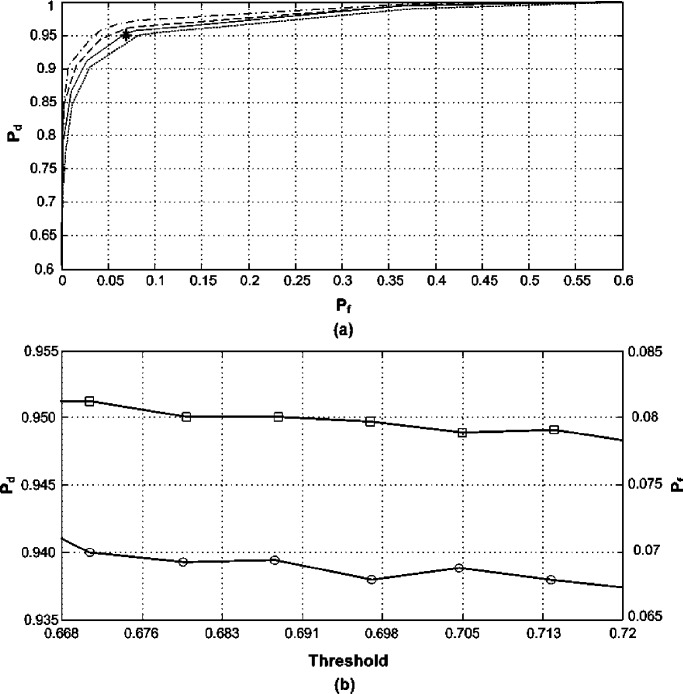
Performance of a system subband. (a) ROC for $K = 13$ (dotted line), $K = {K_{\min}} = 14$ (solid line), $K = 15$ (dashed line), $K = 16$ (dashdot line); asterisk is (0.07,0.95). (b) ${P_d}$ (squares) and ${P_f}$ (circles) for ${\gamma _{\min}} \le \gamma \le {\gamma _{\max}}$ and $K = {K_{\min}}$.

Consider a communication system operating over the frequency range [1.35, 1.45] GHz which is divided into 20 nonoverlapping frequency subbands, 5 MHz each (i.e., $B = 100 {\rm MHz}$ and ${B_{C}} = 5 {\rm MHz}$). The spectrum occupancy is expected to be 10% at most, i.e., ${L_{A}} = 2$ and ${{L_{A}}/ L} = 0.1$. The SNR is $- 0.5 {\rm dB}$. A Hanning window of length 1.25 $\mu {\rm s}$ and an average sampling rate of 90 MHz are used. Whereas, a valid low bandpass uniform sampling rate that would avoid aliasing in the system frequency range is ${f_{US}} = 224 {\rm MHz}$. In the following two subsections, we demonstrate the SARS method with the aid of numerical examples. All the plots in Figs. 2, 5 and 6 were obtained from 10 000 independent experiments.

### A. Example 1: Active Subbands With Equal Power Levels

Here, we examine spectrum sensing with (2) where the present transmissions are BPSK and are of equal power levels. Thus $\mathhat \eta = 1$ is considered in order to countermeasure for any possible decline in the estimator's consistency as discussed in Section III. If we presume that the user demands a probability of false alarm ${P_{f}} \le 0.07$ and that of detection ${P_{d}} \ge 0.95$ for all the system subbands, the required number of estimate averages utilizing (57) is $K \ge 14$, i.e., ${K_{\min}} = 14$. Those probabilities are arbitrarily chosen as an example to depict the behavior of the SARS technique and other probabilities can be selected. Fig. 2(a) shows the ROC plots in one of the system subbands for various $K$ values sweeping across a range of possible threshold values; the asterisk indicates the minimum sought ${P_{d}}$ and maximum permitted ${P_{f}}$. In Fig. 2(b), ${P_{d}}$ and ${P_{f}}$ are displayed for the threshold values determined by (46) and (47) where $K = {K_{\min}}$.

It can be seen in Fig. 2 that the desired system performance was delivered with a sampling rate of 90 MHz. Hence savings of around 60% on the sampling rate and more than 20% on the number of processed samples according to (62) and (63) were attained by using the proposed approach in this paper. At $K = {K_{\min}}$, the acquired probabilities match to a great extend the minimum specified ones. This confirms the reasonable conservativeness of the provided recommendations and that the assumptions undertaken in the conducted analysis did not have noticeable effects on the accuracy of the obtained results. Fig. 2(b) vindicates the effectiveness of the thresholding regime described by (60). If $\mathhat \eta = 0.5$ was chosen, i.e., the impact of signal's cyclostationarity was not recognised, the minimum number of estimate averages would be ${\mathhat K_{\min}} = 10$ which would jeopardize the system response.

**Fig. 3.**
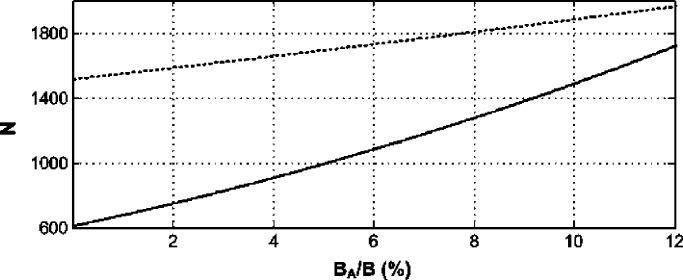
Total number of processed samples for TRS (solid line) and uniform sampling (dotted line).

For illustration purposes, Fig. 3 exhibits the minimum total number of processed samples for uniform sampling and TRS techniques given by (62) and (63) for various spectrum utilizations (assuming constant ${\rm SNR}$), ${P_{f}} = 0.07$ and ${P_{d}} = 0.95$. It is clear that as the spectrum occupancy decreases, i.e., either by fewer subbands being active or extending the monitored bandwidth, the gains of the SARS method become more evident in terms of the total number of processed samples.

### B. Example 2: Active Subbands With Various Power Levels

**Fig. 4.**
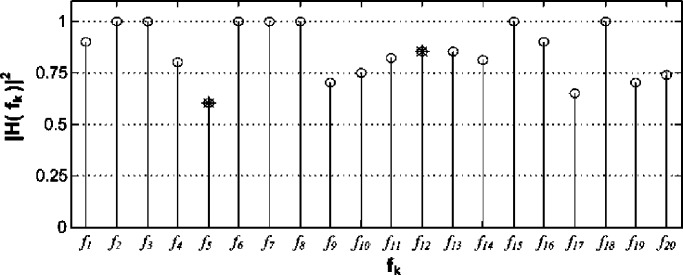
Propagation channel squared magnitude frequency response ${\left\vert {H({f_k})} \right\vert^2}$; the asterisks mark ${\left\vert {H({f_k})} \right\vert^2}$ at the central frequencies of the two examined subbands in Figs. 5 and 6.

**Fig. 5.**
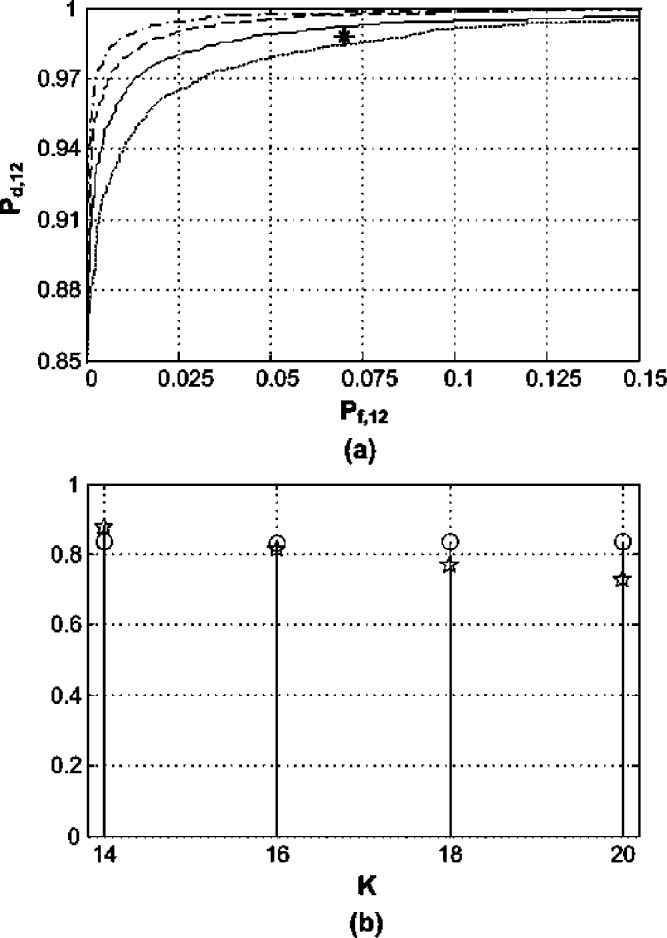
Performance of the targeted subband. (a) ROC for $K = 14$ (dotted line), $K = 16$ (solid line), $K = 18$ (dashed line) and $K = 20$ (dashdot line); asterisk is $\left({{\Delta _{12}},{\ell _{12}}} \right)$. (b) ${m_{1,12}} - {m_{0,12}}$ (circles) and ${Q^{- 1}}\left({{\Delta _{12}}} \right){\sigma _{0,12}} - {Q^{- 1}}\left({{\ell _{12}}} \right){\sigma _{1,12}}$ (stars).

**Fig. 6.**
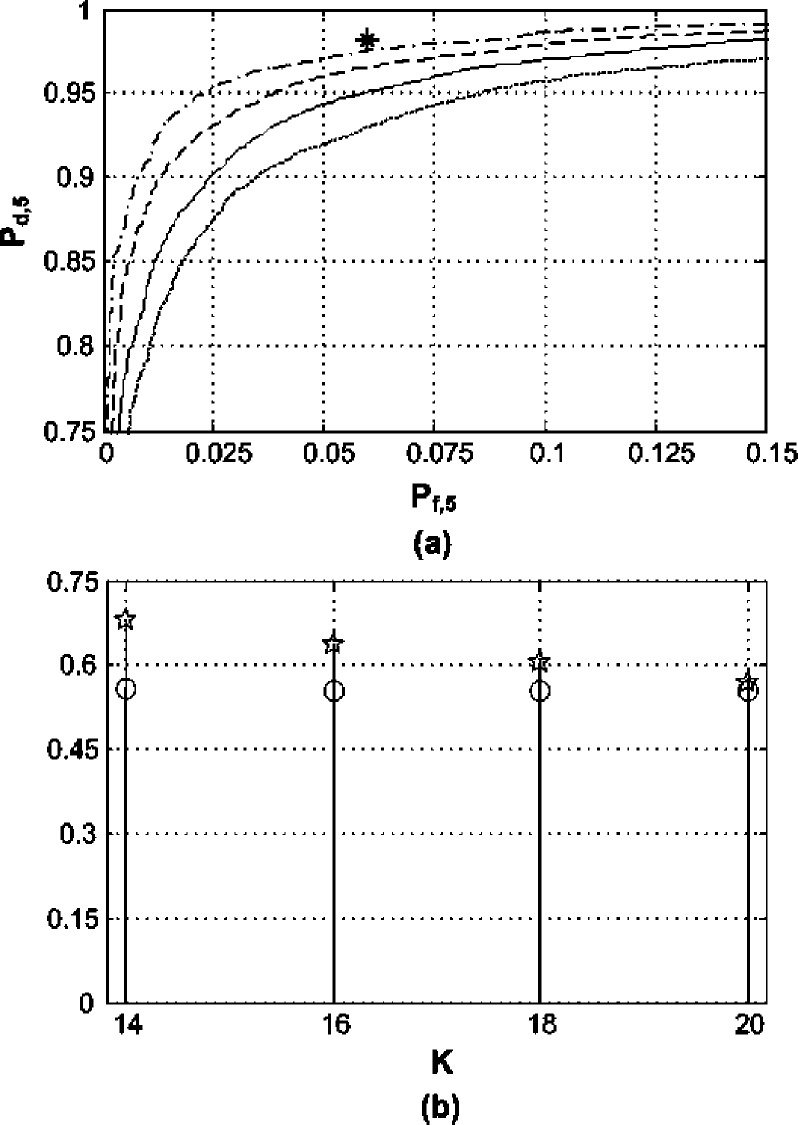
Performance of the subband centered at ${f_5}$. (a) ROC for $K = 14$ (dotted line), $K = 16$ (solid line), $K = 18$ (dashed line) and $K = 20$ (dashdot line); asterisk is $\left({{\Delta _5},{\ell _5}} \right)$. (b) ${m_{1,5}} - {m_{0,5}}$ (circles) and ${Q^{- 1}}\left({\Delta _5} \right){\sigma _{0,5}} - {Q^{- 1}}\left({\ell _5} \right){\sigma _{1,5}}$ (stars).

The transmissions in this example are affected by the propagation channel whose squared magnitude is displayed in Fig. 4. The incoming signal is expected to be a combination of 16 QAM, 256 QAM, and QPSK transmissions. Thus $\mathhat \eta = 0.5$ is chosen and substituted into (57). The aim is to meet the detection requirements of the targeted subband with the central frequency ${f_{12}}$ where ${P_{f,12}} \le 0.07$ (i.e., ${\Delta_{12}} = 0.07$) and ${P_{d,12}} \ge 0.98$ (i.e., ${\ell_{12}} = 0.98$). The minimum number of estimate averages for the targeted subband is $K \ge 16$, i.e., ${K_{\min}} = 16$, as given by (57). At the same time, another subband with the central frequency ${f_{5}}$ has ${P_{f,5}} \le 0.06$ (i.e., ${\Delta_{5}} = 0.06$) and ${P_{d,5}} \ge 0.972$ (i.e., ${\ell_{5}} = 0.972$). The experimental ROC plots along with both sides of the reliability criterion defined by (48) using ${\Delta_{12}}$, ${\ell_{12}}$, ${\Delta_{5}}$ and ${\ell_{5}}$ for each of the two aforementioned subbands is depicted in Figs. 5 and 6 for various estimate averages. The asterisks in Figs. 5 and 6 are $\left({{\Delta_{12}},{\ell_{12}}} \right)$ and $\left({{\Delta_{5}},{\ell_{5}}} \right)$, respectively.

It is clear from Fig. 5 that the pursued probabilities of the targeted subband were acquired by following the derived reliability recommendations. Besides, Fig. 5(b) shows that the condition in (48) is fulfilled for $K \ge 16$. However, this is not the case for the subband centered at ${f_{5}}$; shown in Fig. 6; that demands $\mathhat K \ge 21 \ge {K_{\min}}$ given its ROC probabilities and power level according to (57). This illustrates the compromising involved when a priority subband is specified by the user. To circumvent such cases, the user should survey the requisites for all system subbands and subsequently choose the combined $K$ and $\alpha$ values that would meet all the desired ${P_{f,k}}$ and ${P_{d,k}}$ in ${{\bf P}_{\bf f}}$ and ${{\bf P}_{\bf d}}$ vectors. Figs. 5 and 6 affirm the accuracy and moderation of the derived reliability conditions.

In general, the above numerical examples demonstrate that SARS can notably reduce the required sampling rates to perform wideband spectrum sensing and yet meet the predefined probabilities of detection and false alarm.

## VI. Conclusion

In this paper, a multiband spectrum sensing method that is based on DASP methodology is proposed. It uses a particular randomized sampling scheme along with appropriate processing to conduct reliable detection. This approach eliminates the adverse effect of aliasing that is inherently present when similar signal processing problems are solved with uniform-sampling-based techniques. The sampling rate is no longer related to the total bandwidth of the monitored subbands. In fact, it is shown in the paper that the sampling rate of the introduced spectrum sensing approach can be arbitrarily low. Taking into account the cyclostationary nature of the processed communication signals, the reliability of the sensing procedure is formulated in terms of the average sampling rate, signal observation window $K{T_{0}}$, signal to noise ratio, power levels of the active overseen subbands and the sought system performance. The provided dependability guideline can be employed as a tool to quantify the trade-off between the required sensing time (i.e., signal observation window) and sampling rate in a given scenario.

Comparing to methods based on uniform sampling, the proposed sensing technique offers substantial savings not only on the sampling rate but also on the total number of processed samples. The latter is particularly visible when dealing with scenarios where the occupancy of the monitored subbands is low.

In order to be able to reconstruct the detected signal from the collected samples, it is necessary that the sampling rates do exceed the Landau rate [31], i.e., they should exceed at least twice the total bandwidth of the concurrently active subbands ${B_{A}}$. This condition does not have to be met if spectrum sensing is the only goal. Even if signal reconstruction is to be performed, the SARS technique still offers an important advantage over the uniform-sampling-based detectors. In the case of SARS, the sampling rate has to be proportional to the number of the simultaneously active subbands. Whereas, with uniform sampling the sampling rate has to be proportional to the total monitored bandwidth. This observation prompts researching into algorithms for effective and accurate signal reconstruction from nonuniformly sampled data. This paper serves as an impetus to further research into DASP-based spectrum sensing approaches that deploy randomized sampling schemes other than total random sampling.
